# Mechanical behavior of provisional implant prosthetic abutments

**DOI:** 10.4317/medoral.19958

**Published:** 2014-08-17

**Authors:** Rubén Agustín-Panadero, Blanca Serra-Pastor, Ana Roig-Vanaclocha, Juan-Luis Román-Rodriguez, Antonio Fons-Font

**Affiliations:** 1 DDS, PhD. DDS. DDS. DDS, PhD. MD, DDS, PhD. Occlusion and Prosthodontic Teaching Unit, Department of Dental Medicine, Faculty of Medicine and Dentistry, University of Valencia, Spain

## Abstract

Introduction: Implant-supported prostheses have to overcome a major difficulty presented by the morphology and esthetics of peri-implant tissues in the anterior sector. Diverse therapeutic techniques are used for managing the mucosa adjacent to the implant and the most noteworthy is immediate/deferred fixed provisionalization. Objectives: In vitro testing of strength and deformation of implant prosthetic abutments made from different materials (Titanium/PEEK/methacrylate). Material and Methods: Forty Sweden&Martina® implant prosthetic abutments (n=40) were divided into five groups: Group MP: methacrylate provisional abutments with machined titanium base; Group PP: Poly ether ether ketone (PEEK) provisional abutments; Group TP: titanium provisional abutments; Group TAD: titanium anti-rotational definitive abutments; Group TRD: titanium rotational definitive abutments. Their mechanical behavior under static loading was analyzed. Samples were examined under a microscope to determine the type of fracture produced. Results and Conclusions: Definitive anti-rotational titanium abutments and definitive rotational titanium abutments achieved the best mean compression strength, while PEEK resin provisional abutments obtained the lowest. The group that showed the greatest elastic deformation was the group of titanium provisional abutments.

** Key words:**Immediate loading, immediate provisionalization, implant prosthetic abutment, definitive implant prosthetic abutment.

## Introduction

Esthetics have become an increasingly important issue in contemporary dentistry. In implant dentistry aesthetics are evaluated by a range of parameters including color, shape, whether the definitive prosthesis has a natural appearance and most importantly, the topography and appearance of the soft tissues (1). In this way, esthetic success does not only depend on the prosthesis itself, but is largely determined by the appearance of the soft tissues around it. But soft tissue management in implant dentistry is complex and the esthetic objectives of implant treatments are often difficult to achieve.

At present, a number of techniques can facilitate soft tissue management including the placement of a provisional prosthesis that can be immediate or deferred (2-4). The provisional prosthesis, placed before the definitive prosthesis, allows the tissue to develop more quickly and suggests the definitive gingival shape, and this can be modified in the course of a series of clinical visits until the desired emergence profile is achieved (3,5,6). According to Priest, the placement of a provisional prosthesis is an essential tool for achieving esthetic outcomes for implants located in the anterior sector; a provisional fixed prosthesis (attached to the implant) is the most effective for satisfactory soft tissue management (2). Furthermore, the choice of provisional restoration type can influence esthetics during the integration period and soft tissue healing (1-3). In this way, provisional restorations for one-piece implants have evolved from being a temporary resource during bone and soft tissue integration, to becoming an essential therapeutic tool used to evaluate patient expectations, to facilitate communication with the laboratory and generally optimize the definitive outcomes (1-3,7).

For successful provisionalization, dentists need knowledge and understanding of the different materials and products available on the market and their behavior. Strength, elastic behavior and bond capacity to coverage materials of implant-prosthetic abutments will determine their survival rate in the mouth. So when it comes to choosing one abutment type or another, dentists must assess whether the provisionalization needs to be of short-, medium- or long-term duration.

## Objectives

The objectives of the present study were as follows.

To test the fracture resistance in vitro of different implant prosthetic abutments used to support provisional prostheses.

To measure the deformation of implant-prosthetic abutments after static loading/compression testing.

To evaluate the images of fractures and changes to implant-to-abutment fit after compression strength testing.

To facilitate the selection of abutment type according to the time it must remain in the mouth.

## Material and Methods

1.1 Materials

The mechanical behavior of 40 Sweden&Martina® implant prosthetic abutments was analyzed, subjecting the samples to compression strength testing. The study used 40 Sweden&Martina Khono (Sweden&Martina ® SPA. Due Carrere. Italy) implants with internal hex connections, 4.25 mm in diameter and 11.5 mm long.

Forty screw-retained abutments, of which 24 were provisional and 16 definitive (n=40) were divided into five groups of eight according to material ( Table 1): Group MP: eight methacrylate provisional abutments with machined titanium base; Group PP: Polyether ether ketone (PEEK) provisional abutments; Group TP: titanium provisional abutments; Group TAD: titanium anti-rotational definitive abutments; Group TRD: titanium rotational definitive abutments.

**Table 1:**
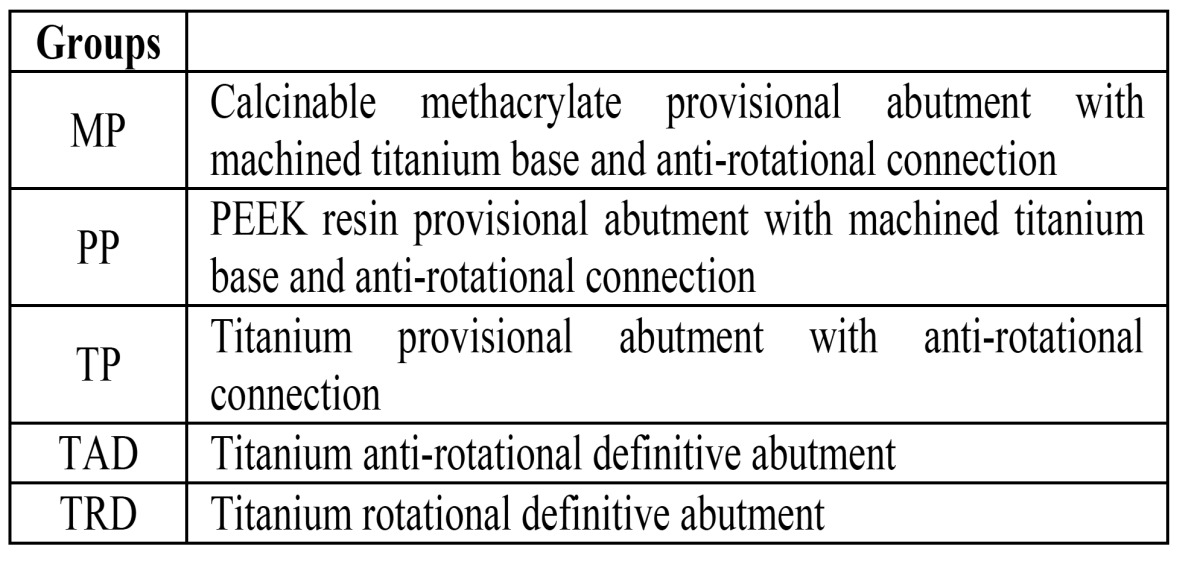
Distribution of groups.

1.2 Sample Preparation 

Implant-cylinder complex

When the study groups and sample sizes had been decided, the specimen/test design was conceived following UNE-ISO 14801 specifications for fatigue testing of single post end osseous dental implants with straight abutments and their prosthetic components, whereby specimens must be angled along an axis that forms a 30º±2º angle to the direction of the force exercised by the test machine. The specification also states that all materials must be used according to the instructions provided by their manufacturers.

Forty implants (n=40) were set in copper cylinders with a diameter of 22 mm. A positioning key was fabricated to ensure the correct angling (30º) of the implant in the cylinder. This key was made using a wax-up of a trip odic structure attached to an implant-supported abutment. The wax structure was then cast in a non-noble metal (Cr-Co). When the key had been fabricated, an implant was screwed to it, applying 15 N/cm2 torque, and the key-implant complex positioned over the cylinder, which was then filled with Exakto-Form epoxy resin (Bredent GmbH & Co. KG. Senden. Germany) to fix the implant in the correct position/angle. When the resin polymerization period had passed (45 minutes, as recommended by the manufacturer), the key was unscrewed from the implant, leaving it set at an angle of 30º.

Conditioning dimensions of implant-prosthetic abutments

To standardize the dimensions of the different abutments, they were milled using a DFM-75E precision micro-milling machine (VOP Ltd. Botevgrad, Bulgaria). In this way, performed abutments of different shape were made uniform. The dimensions were 3 mm diameter by 8 mm long, maintaining the polished titanium collar (1.5 mm height).

Screwing abutments to the cylinder-implant complex 

The milled prosthetic implant abutments were screwed to the implants with an electric prosthetic screwdriver (W&H Dentalwerk, Bürmoos Gmb, Bürmoos, Austria) applying a torque of 30 N/cm2 as recommended by implant manufacturers Sweden&Martina®.

2.Methods

2.1Compression Test 

The compression test was performed with a static load universal test machine (Instron® model 4202, Instron®, Barcelona, Spain) fitted with a load cell of 5000 N.

The force direction was the same for all samples and the load was applied by means of a flat-surfaced antagonist. The load applicator made a vertical motion descending onto the sample, applying a continuous vertical force onto the abutment with a crosshead speed of 0.5 mm/min. The test machine was stopped when it had produced the first abutment fracture and the force provoking the fracture was registered in Newtons (N).

2.2 Deformation Analysis

During compression strength testing, elastic deformation of the abutments at the point of maximum loading was registered. Deformation was interpreted as displacement (in millimeters) of the Instron machine’s load applicator from the initial test position to the moment of abutment failure.

2.3 Failure analysis and Implant-to-Abutment Fit 

Samples were examined under a Leica optical microscope (Leica Microsystems S.L.U. Barcelona, Spain), capturing images of the specimens before compression strength testing. The initial fit between each implant and abutment was examined at x25 enlargement. The process was repeated after testing to evaluate the differences motivated by load transmission from the abutment to the implant connection. An examination was made of each fracture and samples were examined in order to observe any changes to the implant-abutment connection and assess any loss of implant-to-abutment fit.

2.4 Statistical Analysis 

Statistical analysis was performed with SPSS software for Windows (version 14.0, SPSS Inc., Chicago, IL, U.S.A.). Descriptive non-parametric analysis of the load and deformation variables was made (mean, standard deviation, range and median). The Mann-Whitney test was applied to identify differences between groups, two by two, for both variables. Statistical significance was established at 5% (*p*<0.05).

## Results

3. Compression Test 

3.1 Compression Strength Data 

Group TAD (titanium anti-rotational definitive abutments) achieved the greatest compression strength, with a mean fracture resistance of 1106.7±344.4 N. The group presenting the lowest resistance was Group PP (PEEK resin provisional abutments), with a mean fracture resistance of 329.4±103.6 N. Group TP (titanium provisional posts) showed the second highest values, with a mean fracture value of 985.4±350.3 N. Group TRD (titanium definitive abutments with rotational connection) was in third place with a mean fracture value of 853.3±409 N. In penultimate place, Group MP (methacrylate provisional abutments with machined titanium base) presented a mean of 370.7±137.8 N.

**Figure 1:**
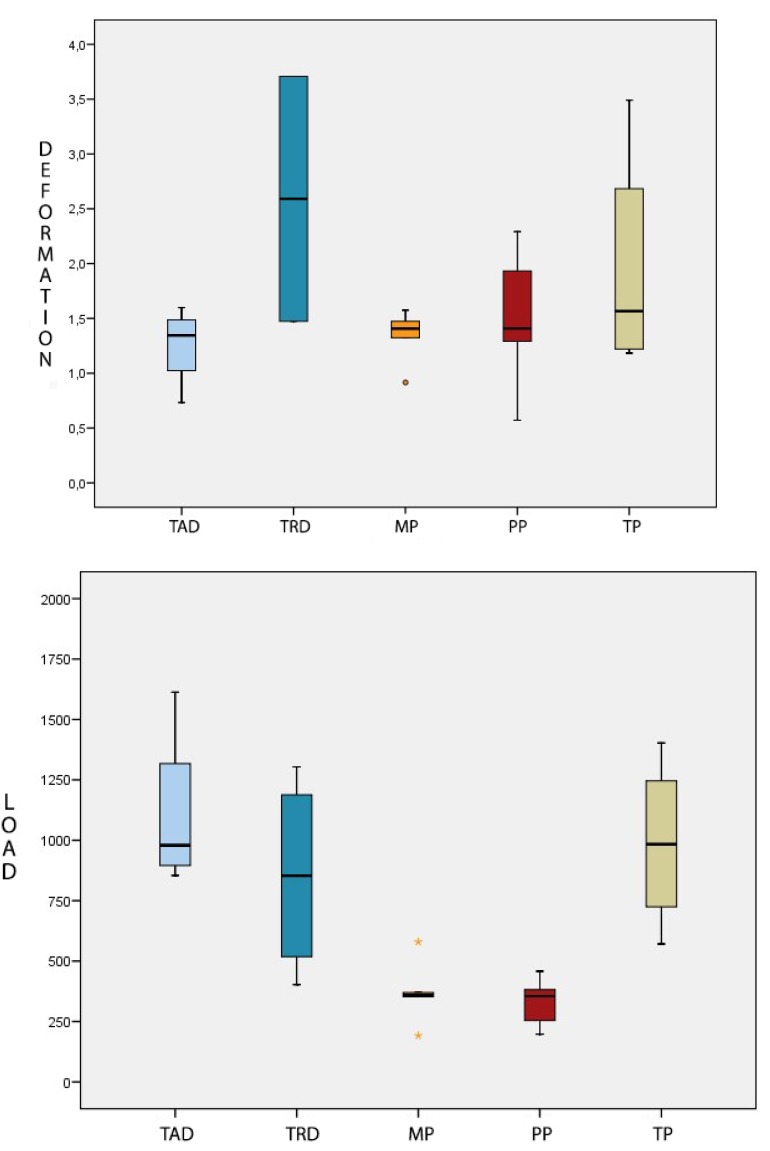
A) Box-plot of load values (N) obtained for each group. B) Box-plot of deformation values (mm) obtained for each group.

Fig. 1 A, a box plot, shows the variations in fracture resistance between groups.

Applying the Mann-Whitney test ( Table 2), it was found that: Group MP was less resistant than Groups TAD and TRD, with statistically significant difference. Group PP abutments were less resistant than Groups TAD and TRD, with statistically significant differences. Group TP was seen to be significantly more resistant than Groups MP and PP.

**Table 2:**
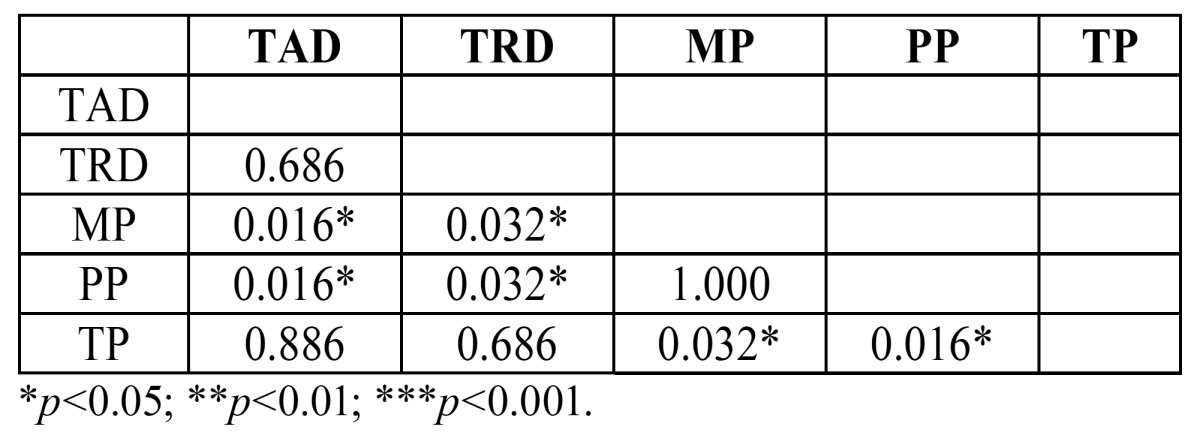
Mann-Whitney Test applied to identify statistically significant differences in resistance to static loading between groups.

3. 2. Deformation Analysis

Group TP (titanium provisional abutments) suffered the most deformation with a mean of 1.952±1.072 mm. Group TAD (titanium anti-rotational definitive abutments) underwent the least deformation with a mean of 1.256±0.369 mm. In second place, Group TRD (titanium definitive abutments with rotational connection) suffered mean deformation of 1.532±0.59 mm. Group PP (PEEK resin provisional abutments) followed with a mean value of 1.499± 0.657 mm. Group MP was the second least deformable group with a mean deformation value of 1.339±0.254 mm (Fig. 1 B).

However, the Mann Whitney test did not identify statistically significant differences between the groups (*p*=0.619) ( Table 3).

**Table 3:**
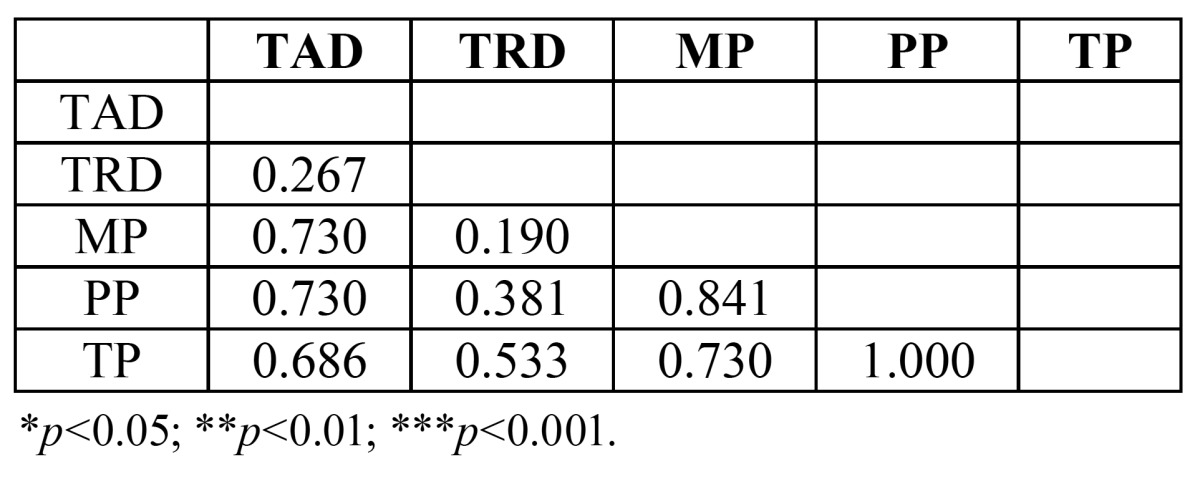
Mann-Whitney Test applied to identify statistically significant differences in deformation between groups.

**Figure 2:**
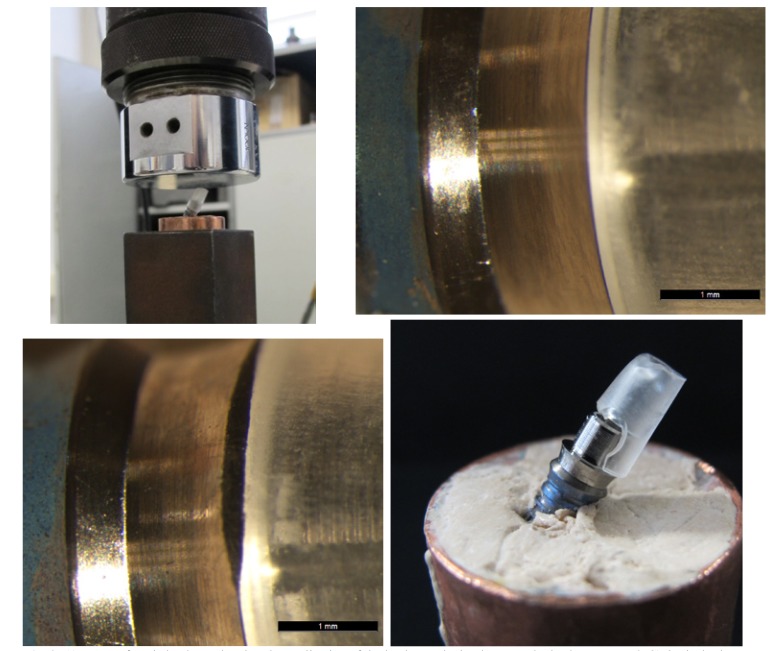
A) Image of static load test showing the application of the load onto the implant-prosthetic abutment. B&C) Optical microscope images of condition of a Group MP abutment pre- and post-loading. No differences were registered at the implant-abutment union. D) Image of Group MP abutment showing the methacrylate fracture at the interface with the machined titanium base.

3. 3 Failure Analysis and Implant-to-Abutment Fit For Group MP, all fractures occurred between the methacrylate and the machined titanium base, separating the two parts. When implant-abutment fit was examined, no differences in fit between pre- and post-loading were observed (Fig. 2 A, B & C).

**Figure 3:**
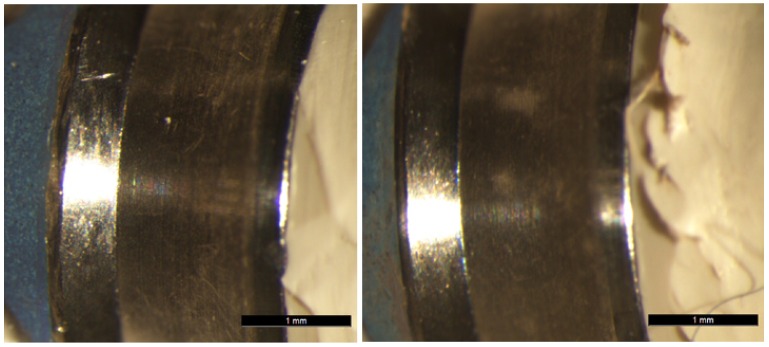
A&B). Optical microscope images of condition of a Group PP abutment pre- and post-loading. No differences were registered at the implant-abutment union.

Group PP abutments all fractured at the union between the PEEK resin and the titanium base, causing the two structures to separate as in Group MP. No differences in the initial and posttest fit were observed; the initial perfect implant-to-abutment fit remained intact (Fig. 3 A & B).

**Figure 4:**
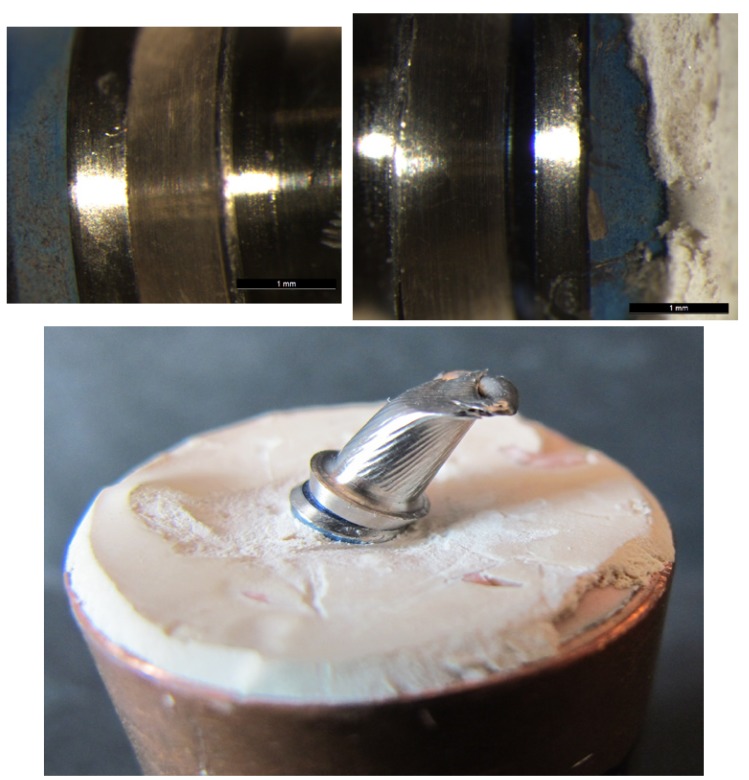
A, B&C) Optical microscope images of condition of a Group TP abutment pre- and post-loading. A separation at the implant-abutment union can be observed after the static load test.

Group TP abutments did not suffer fracture but there was some deformation in the most coronal area. Microscope examination revealed changes to the initial fit after loading, whereby loading provoked a loss of fit at the implant-abutment union with deformation and elongation of the fixing screw (Fig. 4 A, B & C).

**Figure 5:**
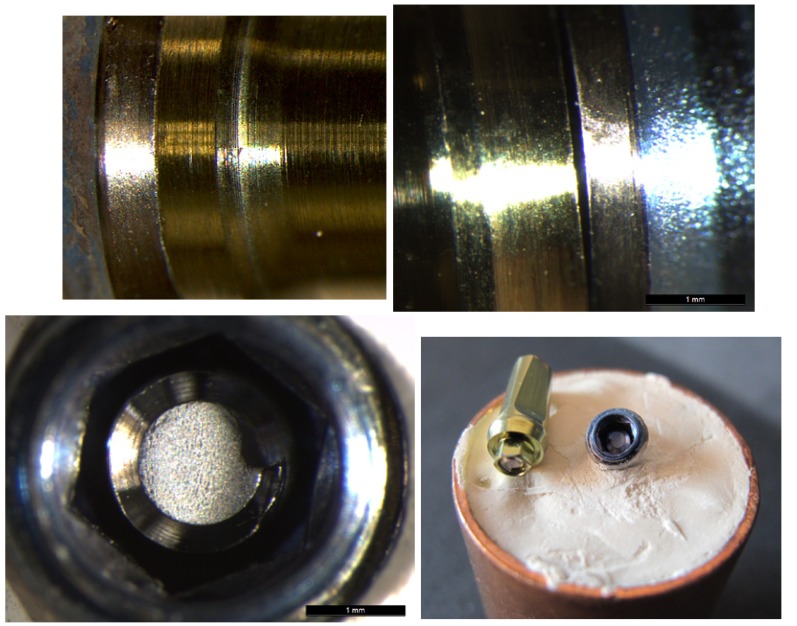
A&B) Optical microscope images of condition of a Group TAD abutment pre- and post-loading. A separation at the implant-abutment union can be observed after the static load test. C&D). Image of one Group TAD specimen showing the fractured screw left inside the implant after static load testing.

No fractures occurred in Group TAD. There was some deformation of the fixing screw, which fractured in two cases and was deformed in a further three, provoking their elongation. In this way, when the screw did not fracture, the samples suffered a loss of fit at the implant-abutment union; when the screw fractured, the broken part remained in the implant (Fig. 5 A, B, C & D).

**Figure 6:**
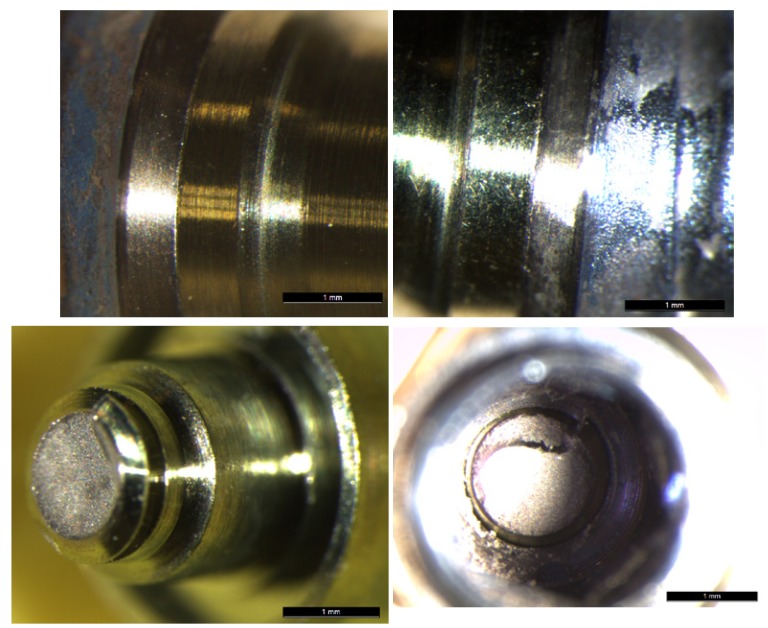
A&B) Optical microscope images of condition of a Group TRD abutment pre- and post-loading. No differences were registered at the implant-abutment union. C&D) Image of one Group TRD specimen showing a fractured rotational definitive abutment provoked by static loading.

For Group TRD abutments, as in the previous group, the abutment structure remained intact, but loading had provoked deformation of the fixing screw. For three samples, the screw fractured and for another two the screw was deformed. For those samples whose screw had not fractured, no differences in implant-to-abutment fit were observed under microscope examination. (Fig. 6 A & B) However, when the screw fractured, this occurred at a more apical point than in the previous group as with this design the screw forms part of the abutment structure (Fig. 6 C & D).

## Discussion

Although provisional implant-supported resin crowns are expected to function in the oral environment only for a short period of time ranging from 2 weeks to 3 months, they must be able to resist occlusal forces during function. Depending on the duration or complexity of the surgical or reconstructive implant therapy, temporary restorations may function for even a longer period of time. The literature provides important data as to the forces that implant-supported prosthetics must withstand in the different sectors of the mouth under normal conditions. According to Ferrario, the occlusal load that a single tooth must withstand in the anterior region is 150 N (8). In this in vitro study, all the abutments tested fulfilled the load requirements for survival.

4.1 Discussion of Materials and Methods 

4.2 Sample Design

Sample design in the present study followed the geometry specified in ISO 14801 for testing end osseous dental implants, with the implant set at an angle of 30º to the test machine`s load cell. This setup was used in the majority of articles reviewed in preparation for this study (9-15).

The material in which the implants were set, epoxy resin, was selected due to its elastic modulus of 3 GPa, which meets the requirements of ISO 14801 and also because this material was used in the majority of studies comparable to the present one (9-15).

4. 3 Compression Test

The type of test and test design, in this case a compression strength test, was based on ISO regulations for studies of end osseous dental implants (ISO 14801:2007). Furthermore, a number of authors have proposed similar test designs (10-14); the crosshead speed of 0.5 mm/min was used in the majority of comparable studies (9-15).

4. 4 Discussion of the Results 

To date, little information is available in the literature on the survival rate of provisional implant-supported restorations; only one article has been published that studied provisional abutments supporting single crowns (16). No scientific data has been published for the strength of provisional abutments on implants before prosthetic restoration placement.

The present study tested fracture resistance of abutments of different types and materials, including two groups of definitive abutments, which are often used to support provisional prosthetics. Other studies have also used static loading to test definitive abutments (11,14-16). In a study of different implant-supported provisional restoration types, Santing *et al*. concluded that provisional crowns bonded onto PEEK abutments at central incisor sites presented a much lower resistance to fracture (95±21N) than crowns bonded to titanium abutments (1009±94N); these findings concur with the present study, which also found that PEEK resin abutments showed the lowest fracture resistance values (329.4±103.6N) (16). Steinebrunner (2008), in a study evaluating fracture resistance of different implant-to-abutment connections, obtained a mean resistance of 780 N for anti-rotational titanium abutments (Zimmer Dental) tested under static loading (15). Truninger (2008) studied the fracture resistance of zirconia abutments, using titanium abutments with rotational internal connections (Straumann) as a control group; mean fracture values obtained were 714 N for the titanium abutments (11). Sannino *et al*. (2013), made a static load assay of titanium abutments with anti-rotational connections (Leone®,) obtaining a mean fracture value of 906 N (14). In the present study, the titanium abutment groups obtained the highest facture resistance values (853.3±409N and 1106.7±344.4N).

Regarding the elastic deformation of different types of provisional abutment, the present study data cannot be compared with any other, as no other scientific research on this topic has been published.

## Conclusions

Although an in vitro study will always have limitations, it may be concluded that.

1. The PEEK resin and methacrylate provisional abutment groups showed less fracture resistance than the titanium abutment groups.

2. The group that showed the greatest elastic deformation was the group of titanium provisional abutments.

3. Titanium provisional abutments showed high fracture resistance; however, their structure deformed badly and samples suffered a loss of fit at the implant-abutment union.

4. On the basis of the present results, the use of PEEK resin or methacrylate abutments is recommended when the provisional fixed prosthesis is to remain in the mouth for one to three months. For mid-term provisional prosthetics (three to six months), titanium provisional or definitive rotational abutments are recommended. When provisional prostheses are to remain in the mouth for longer periods (six to twelve months) anti-rotational titanium definitive abutments are recommended.
